# Diagnostic Yield of Transbronchial Lung Cryobiopsy Compared to Transbronchial Forceps Biopsy in Patients with Sarcoidosis in a Prospective, Randomized, Multicentre Cross-Over Trial

**DOI:** 10.3390/jcm10235686

**Published:** 2021-12-02

**Authors:** Maik Häntschel, Ralf Eberhardt, Christoph Petermann, Wolfgang Gesierich, Kaid Darwiche, Lars Hagmeyer, Thomas V. Colby, Falko Fend, Dirk Theegarten, Hanns-Olof Wintzer, Michael Kreuter, Werner Spengler, Annika Felicitas Behrens-Zemek, Richard A. Lewis, Henry C. Evrard, Ahmed Ehab, Michael Böckeler, Jürgen Hetzel

**Affiliations:** 1 Department of Medical Oncology and Pneumology, Eberhard Karls University of Tübingen, 72074 Tübingen, Germany; werner.spengler@med.uni-tuebingen.de (W.S.); annika-behrens@gmx.net (A.F.B.-Z.); dr.a.ehab@gmail.com (A.E.); m.boeckeler@medius-kliniken.de (M.B.); juergen.hetzel@med.uni-tuebingen.de (J.H.); 2 Department of Internal Medicine—Pneumology, Cantonal Hospital Winterthur, 8400 Winterthur, Switzerland; 3 Department of Pneumology and Critical Care Medicine, Asklepios-Klinik Barmbek, 22307 Hamburg, Germany; r.eberhardt@asklepios.com; 4 Department for Pulmonary Diseases, Asklepios-Klinik Hamburg, 22307 Hamburg, Germany; c.petermann@asklepios.com; 5 Comprehensive Pneumology Center Munich, Asklepios-Fachkliniken Munich-Gauting, 81377 Munich, Germany; w.gesierich@asklepios.com; 6 Department of Interventional Pneumology, Ruhrlandklinik—University Medicine Essen, University of Duisburg-Essen, 47057 Duisburg, Germany; kaid.darwiche@rlk.uk-essen.de; 7 Clinic for Pneumology and Allergology, Center of Sleep Medicine and Respiratory Care, Bethanien Hospital, 42699 Solingen, Germany; lars.hagmeyer@klinik-bethanien.de; 8 Department of Pathology (Emeritus), Mayo Clinic, Scottsdale, AZ 13400, USA; colby.thomas@mayo.edu; 9 Institute of Pathology and Neuropathology, Reference Center for Hematopathology University Hospital, Eberhard Karls University of Tübingen, 72074 Tübingen, Germany; Falko.Fend@med.uni-tuebingen.de; 10 Institute of Pathology, University Hospital Essen, University of Duisburg-Essen, 7057 Essen, Germany; dirk.theegarten@uk-essen.de; 11 Institute for Pathology, MVZ Hanse Histologikum, 22547 Hamburg, Germany; wintzer@pathologie-hh.de; 12 Department of Pathology/Hematopathology, Institute for Hematopathology, 22547 Hamburg, Germany; 13 Center for Interstitial and Rare Lung Diseases, Department of Pneumology, Thoraxklinik, University of Heidelberg and German Center for Lung Research (DZL), 69117 Heidelberg, Germany; kreuter@uni-heidelberg.de; 14 University of Worcester, Worcester WR2 6AJ, UK; lewisr@doctors.org.uk; 15 Department Physiology of Cognitive Processes, Max Planck Institute for Biological Cybernetics, 72076 Tübingen, Germany; henry.evrard@tuebingen.mpg.de; 16 Center for Integrative Neuroscience, Eberhard Karls University of Tübingen, 72074 Tübingen, Germany; 17 Center for Biomedical Imaging & Neurostimulation, Nathan Kline Institute for Psychiatric Research, Orangeburg, NY 10962, USA; 18 Department of Pneumology, Klinik Loewenstein, 74245 Loewenstein, Germany; 19 Chest Medicine Department, Mansoura University, Mansoura 35516, Egypt; 20 Department of Internal Medicine, Gastroenterology and Tumor Medicine, 73760 Ostfildern-Ruit, Germany

**Keywords:** sarcoidosis, bronchoscopy, transbronchial biopsy, cryobiopsy

## Abstract

Background: Transbronchial lung forceps biopsy (TBLF) is of limited value for the diagnosis of interstitial lung disease (ILD). However, in cases with predominantly peribronchial pathology, such as sarcoidosis, TBLF is considered to be diagnostic in most cases. The present study examines whether transbronchial lung cryobiopsy (TBLC) is superior to TBLF in terms of diagnostic yield in cases of sarcoidosis. Methods: In this post hoc analysis of a prospective, randomized, controlled, multicentre study, 359 patients with ILD requiring diagnostic bronchoscopic tissue sampling were included. TBLF and TBLC were both used for each patient in a randomized order. Histological assessment was undertaken on each biopsy and determined whether sarcoid was a consideration. Results: A histological diagnosis of sarcoidosis was established in 17 of 272 cases for which histopathology was available. In 6 out of 17 patients, compatible findings were seen with both TBLC and TBLF. In 10 patients, where the diagnosis of sarcoidosis was confirmed by TBLC, TBLF did not provide a diagnosis. In one patient, TBLF but not TBLC confirmed the diagnosis of sarcoidosis. Conclusions: In this post hoc analysis, the histological diagnosis of sarcoidosis was made significantly more often by TBLC than by TBLF. As in other idiopathic interstitial pneumonias (IIPs), the use of TBLC should be considered when sarcoidosis is suspected.

## 1. Introduction

The sampling technique used for histological diagnosis is usually determined by the localization of the characteristic pathology within the organ. In interstitial lung diseases (ILDs) where there is predominant involvement of the lung parenchyma, transbronchial cryobiopsy (TBLC) has been shown to be superior to transbronchial forceps biopsy (TBLF) in providing diagnostic material [1]. Consequently, TBLC has become routinely used for the diagnosis of suspected idiopathic pulmonary fibrosis (IPF) [2] and hypersensitivity pneumonitis [3], among others.

In contrast, TBLF is generally considered to be of value in bronchocentric pathology—sarcoidosis being the archetypical example—and is used as a standard technique in such diseases [4]. However, a variable diagnostic yield of 40–90% for TBLF alone has been reported, associated with a pneumothorax rate of 1–9% [5,6,7,8,9].

Currently, in most centres, the diagnosis of sarcoidosis is made with endobronchial ultrasound-guided transbronchial needle aspiration (EBUS-TBNA) of lymph nodes, sometimes in combination with endobronchial mucosal biopsy [10]. In some cases, however, a transbronchial biopsy is also required if the other procedures are not diagnostic. The need for histopathologic evidence of sarcoidosis is justified by the possible differential diagnoses such as hypersensitivity pneumonitis, pulmonary infections, and malignant neoplasms. In addition, it is often necessary to increase the level of diagnostic certainty by a tissue sample in order to justify a long-term and potentially side-effect-laden therapy for sarcoidosis. In such cases, TBLF is currently the diagnostic standard.

Considering the known superiority of TBLC over TBLF in the diagnosis of ILDs, the question arises as to whether TBLC may be at least equipotent to TBLF in the diagnosis of bronchocentric disease such as sarcoidosis, or even considered superior. Retrospective data showed a diagnostic yield for TBLC alone, ranging from 66.7% up to 92.6%, which is comparable to TBLF [11,12].

To examine the diagnostic value of TBLF and TBLC in sarcoidosis, we used a subset of data from our large study comparing biopsy-associated bleeding incidence [13]. This large study also evaluated the diagnostic yield of TBLF and TBLC in the subgroup of patients in whom the histological diagnosis was sarcoidosis.

## 2. Materials and Methods

### 2.1. Study Population

We performed a post hoc analysis of our prospective, randomized, multicentre study in six pulmonary centres in Germany [13].

### 2.2. Inclusion Criteria

Patients over 18 years of age with suspected ILD on radiological evaluation and the need for a histological diagnosis. All patients gave their informed consent.

### 2.3. Exclusion Criteria

Patients with any bleeding disorder (international normalized ratio (INR) > 1.3, partial thromboplastin time (PTT) above normal, thrombocytopenia < 100,000/µL), treatment with thienopyridines, oxygen saturation below 90% with a maximum oxygen delivery of two litres per minute, severe cardiac disease, or known pulmonary hypertension above 50 mmHg were excluded.

### 2.4. Data Acquisition

Demographic data of the patients included age, sex, weight, height, smoking status, intake of acetylsalicylic acid, and any other medications.

### 2.5. Bronchoscopy

Bronchoscopy was undertaken in each centre using the local standard practice, which in all cases included the use of a Rusch Bronchoflex tube or a rigid bronchoscope, through which a flexible bronchoscope was inserted to obtain the transbronchial biopsies. No prophylactic balloon placement was performed.

### 2.6. Tissue Sampling

**Figure 1:**
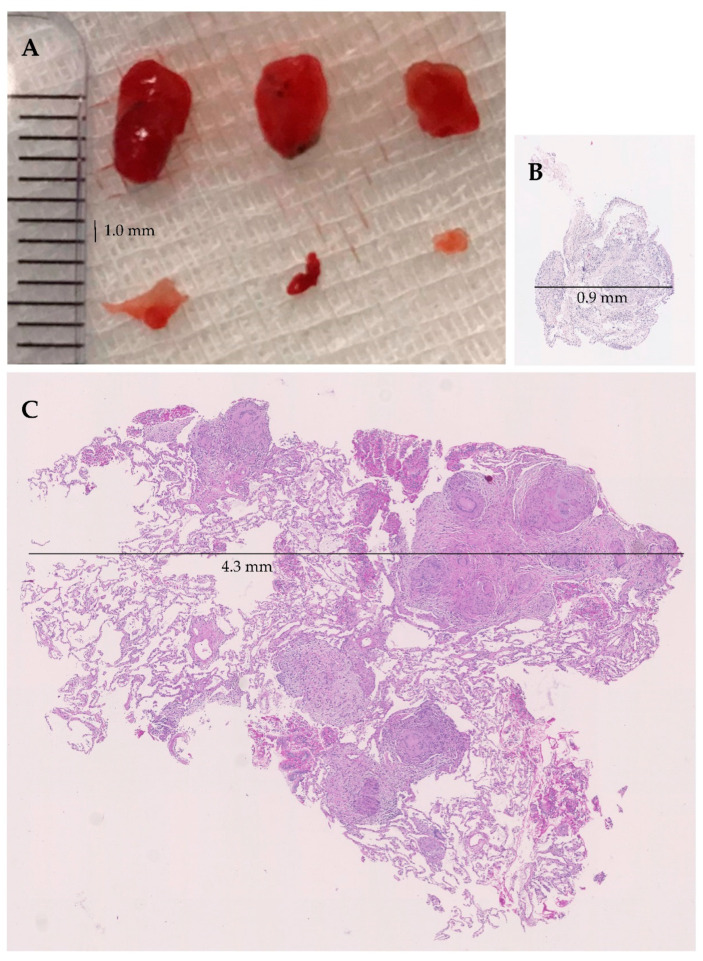
Direct comparison of the dimensions of TBLC and TBLF. (**A**) Macroscopic image of 3 TBLCs (top row, probe size 1.7 mm, freezing time 5 and 6 s) and 3 TBLFs (bottom row, forceps 2.2 mm) (scale in mm); (**B**) Microscopic image (2.5× magnification) of TBLF—diameter 0.9 mm; (**C**) Microscopic image (2.5× magnification) of TBLC—diameter 4.3 mm.

In each patient, both TBLF and TBLC samples were obtained in a randomized order. Fluoroscopic guidance was used for subpleural probe positioning in the majority of cases. TBLC was performed as previously described [14], using reusable cryoprobes of 1.9 or 2.4 mm diameter (Erbe Elektromedizin GmbH, Tübingen, Germany) with a freezing time of 3 to 7 s depending on the freezing power and diameter of the probe. For TBLF, forceps of a diameter between 1.8 mm and 2.6 mm were used. Figure 1 illustrates the size differences between TBLC and TBLF.

### 2.7. Randomization

The sequence of biopsy techniques (TBLF or TBLC) was randomized using consecutive numbered envelopes provided by the Institute of Epidemiology and Medical Biometry, Ulm University, Ulm, Germany. Adherence to randomization was confirmed at the end of the study.

### 2.8. Pathological Evaluation

Pathological evaluations of all tissue samples, which were formalin-fixed, paraffin-embedded, and routinely processed, were undertaken by the cooperating pathology institute in each centre. An independent histological evaluation of all slides was undertaken at the University of Tübingen, where slides were scanned at 0.22 μm/pixel resolution using a slide scanner (AxioScan.Z1; Carl Zeiss GmbH, Göttingen, Germany) equipped with a high-end 3CCD progressive scan colour camera (HV-F202SCL; Hitachi, Marunouchi, Tokyo, Japan) at the Department of Physiology of Cognitive Processes, Max Planck Institute for Biological Cybernetics. The slides themselves were kept for direct visual analyses if necessary. All scanned slide files were randomized to exclude evaluation bias, which could occur if tissue samples obtained by both techniques were analysed consecutively. For study purposes, and to exclude inter-individual bias, pathological evaluation was performed by a single ILD expert pathologist (T.V.C.). Evaluation categorized specimens as ‘diagnostic’ or ‘non-diagnostic’, and if ‘diagnostic,’ then the confidence level of diagnosis (high, low, or no evidence of sarcoidosis) was provided for the entire tissue sample for each technique.

### 2.9. Evaluation of Adverse Effects

Any bleeding during TBLC or TBLF was documented. As several biopsies were taken by each technique in each subject, the most severe bleeding for each technique was determined semi-quantitatively on a four-level scale as previously described [13] with ‘no bleeding’, ‘mild’ (suction alone), ‘moderate’, (additional intervention), or ‘severe’ (prolonged monitoring necessary or fatal outcome) for each intervention. ‘No’ or ‘mild’ bleeding was categorized as clinically irrelevant; moderate and severe bleeding were categorized as clinically relevant.

Exemplary comparison of transbronchial cryobiopsies (TBLC) and forceps biopsies (TBLF) with evidence of sarcoidosis-typical granulomas.

### 2.10. Primary and Secondary Objectives

The primary objective was the histological diagnosis consistent with sarcoidosis (i.e., non-necrotizing granulomatous inflammation compared to sarcoidosis; absence of identifiable microorganisms) from biopsy tissue obtained by TBLC or TBLF. Secondary objectives were: absolute biopsy number, total area of all extracted tissue specimens by either technique in each patient (including the total area of tissue per biopsy), and the incidence and severity of biopsy-associated bleeding.

### 2.11. Statistics

McNemar’s test for the analysis of cross tables and Wilcoxon’s test for the comparison of groups were used. A *p*-level below 5% was regarded as significant.

### 2.12. Approval and Registration

The study design and protocol were approved by the Ethics Committees of Tuebingen (Reference number 035/2011MPG23) and confirmed by each individual site ethics committee. The study was registered with clinicaltrials.gov (NCT01894113).

## 3. Results

**Figure 2:**
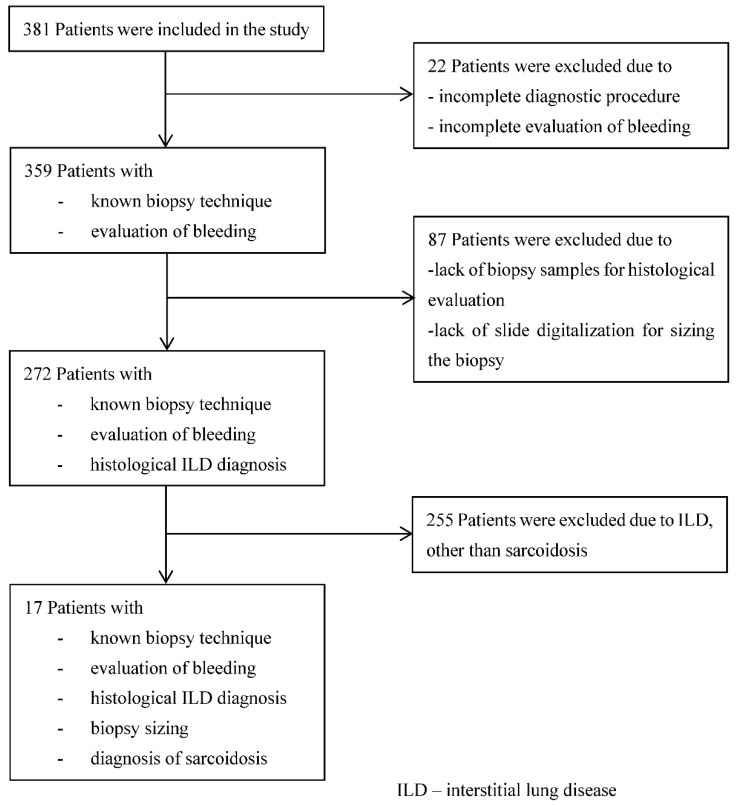
Study population.

A total of 381 patients were included from six German pulmonary centres as previously described [13]. A total of 272 cases with known biopsy technique and established histopathologic diagnoses were evaluated for peri-interventional haemorrhage and biopsy size. A histological diagnosis compatible with sarcoidosis was made in 17 patients. This cohort was used as the basis for evaluating the primary outcome in this study (Figure 2).

Patients (nine women, eight men) had a mean age of 44.6 ± 12.7 years, weight of 80.8 ± 16.0 kg, and height of 173 ± 13 cm. Nine patients (52.9%) were non-smokers, four ex-smokers (23.6%), and four smokers (23.6%). Bronchoscopic intervention data are shown in Table 1.

**Figure 3:**
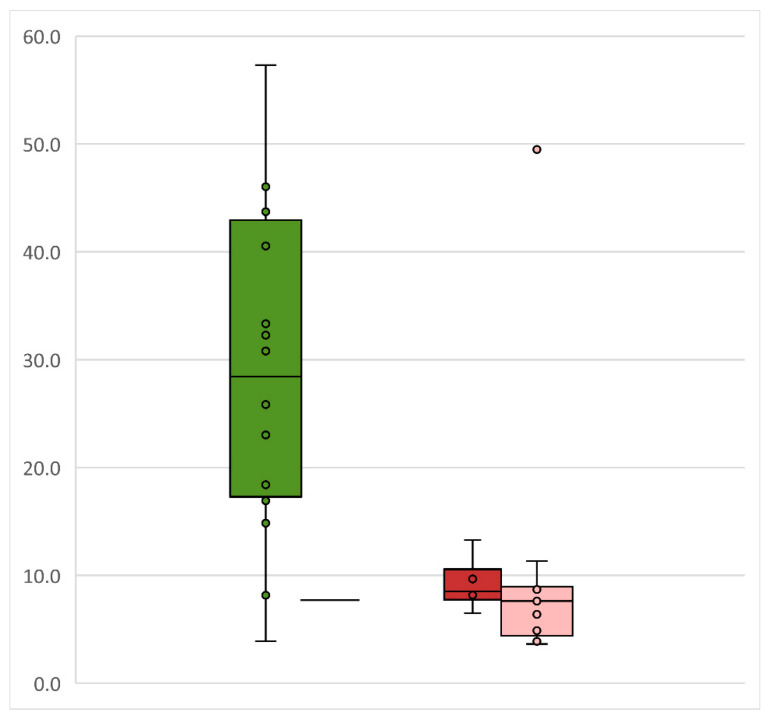
Total biopsy size by biopsy technique and diagnostic value.

In those cases where a sarcoidosis diagnosis was made, there were no significant differences between the total number of biopsies taken, sites of biopsy, size of forceps, or probe used (small/large), nor in the duration of the procedure between TBLC and TBLF, or a difference in ease of positioning the biopsy probe (Table 1). However, the median biopsy size was more than 3-fold larger for the total area of all biopsies (26.07 vs. 8.19 mm^2^), and for the median overall area per biopsy (8.11 vs. 1.72 mm^2^), with TBLC compared to TBLF (Table 1, Figure 3). TBLC produced significantly fewer artefacts than TBLF with no artefacts in 76.5% of TBLCs, compared to only 23.5% of TBLFs (Table 1). The relationship between individual biopsy size and diagnostic yield for sarcoid patients and the diagnostic yield compared to biopsy size is shown in Figure 3, confirming the utility of the larger biopsy size provided by TBLC in producing a diagnostic sample.

In the overall cohort of 272 patients, histological findings consistent with sarcoid were found in 5.9% by TBLC compared to 2.6% by TBLF. There were 10 cases where diagnosis was only made by TBLC, and 1 case where diagnosis was only made only by TBLF (*p* < 0.0001). In six cases, the diagnosis of sarcoid was made by both TBLC and TBLF (Table 2).

Box plot illustrating total biopsy area in mm^2^. On the left side, diagnostic TBLC cases (diag.—dark green box) and a single non-diagnostic TBLC case (non diag.—horizontal line for median/ single case) are shown. On the right side, diagnostic TBLF cases (diag.—dark red box) and non-diagnostic TBLF cases (non-diag.—light red box) are shown. Each dot represents on single case. One case of diagnostic TBLC with a total biopsy area of 207.9 mm^2^ cannot be displayed due to the dimension but is represented in the median line. Horizontal line—median; box size for Quartile 1 and 3. TBLC—transbronchial lung cryobiopsy; TBLF—transbronchial lung forceps biopsy.

### Adverse Events

Numbers of cases with ‘no’ or ‘mild’ bleeding were not significantly different between TBLC and TBLF. Moderate to severe bleeding occurred in three cases with TBLC and none with TBLF (Table 3). No patient experienced serious complications after either TBLC or TBLF, and intensive care or resuscitation were never required.

## 4. Discussion

Transbronchial forceps biopsy is the recommended biopsy technique in the workup of sarcoidosis. However, in some cases, TBLF does not yield a diagnosis. Therefore, TBLC might be of interest also in sarcoidosis. We show here that significantly more cases of sarcoidosis could be diagnosed by TBLC compared to TBLF, and it would appear that this is due to the significantly increased biopsy size that is provided by TBLC compared to TBLF. However, this comes with the increased risk of moderate to severe bleeding, as has already been shown in a previous study [13].

In general, the diagnostic value of bronchoscopic lung biopsies is highly dependent on the biopsy technique used, but also appears to vary between different forms of ILDs. TBLC has been confirmed to be of value in the diagnosis of ILDs [15,16,17] and has consequently been included in guidelines for the diagnosis of idiopathic pulmonary fibrosis [2] and hypersensitivity pneumonia [3], whereas TBLF has not been considered to provide sufficient diagnostic yield to be of value in these diseases. In contrast, TBLF is considered as a standard technique in the diagnosis of bronchocentric diseases such as sarcoidosis and is therefore recommended for this purpose in guidelines [4].

Due to the peribronchial accentuation, TBLF provides a higher diagnostic yield in sarcoidosis compared to other ILDs [18,19,20,21]. TBLF is useful to detect distinctive and often diagnostic findings in small biopsies (such as organisms or neoplasms) [13]. However, the diagnostic value of TBLF alone has been reported to be highly variable and sometimes limited in sarcoidosis [5,6,7,8,9]. Even the combination of TBLF, endobronchial biopsy of the mucosa and fine needle aspiration of lymph nodes may fail to confirm the diagnosis of sarcoidosis in some cases [22,23,24]. In addition, with only cytologically preserved material, gene expression profiling is not always possible. Gene profiling has the future potential of guiding therapy in a similar manner to that currently used in advanced bronchial carcinoma [25].

The larger sample size provided by TBLC not only produces a higher diagnostic yield as clearly shown in this study but may also provide sufficient material for additional gene profiling and thus avoid the need for surgical lung biopsy [26,27]. 

The absence of artefact in the biopsy specimen also contributes to the ability to provide a diagnosis in most settings, although in the case of non-caseating granulomatous disease this may be of less importance.

It is probable that the higher diagnostic value of TBLC in sarcoidosis observed here is explained solely by the increased probability of finding these characteristic features in significantly larger biopsies, but sampling issues remain with both techniques, as evidenced by the case with non-diagnostic TBLC and a diagnostic TBLF.

It is clear therefore from this study that ‘size matters,’ not only in diseases diffusely involving lung parenchyma, but also those with bronchocentric distribution, and the balance needs to be made between the optimal size for diagnosis and the risk of bleeding. We have clearly demonstrated that TBLC is the method of choice for providing diagnostic material in cases of suspected sarcoidosis where a definitive histological diagnosis can be of importance, particularly in those cases with long-term therapeutic consequences. However, TBLC should be undertaken in a unit with experience of both the technique and complication management due to the increased bleeding risk [28]. As prophylactic balloon placement was not used in this study, the bleeding rate of TBLC according to the current standard of care [14,28,29] with prophylactic bronchus blocker is likely to be overestimated.

There are also some limitations to this study. This is a post hoc analyses of a larger study on the role of TBLC in ILD, and this study was not designed to prospectively evaluate the diagnostic yield in sarcoidosis specifically. However, there was a clear medical unmet need to gain further insights into the role of TBLC in sarcoidosis. Furthermore, the number of patients is comparatively small. However, it must be considered that both techniques were used in each of the 17 cases. In addition, the intraindividual comparison allowed identical conditions for both techniques and thus increased the value of direct comparison. Other techniques for the evaluation of sarcoidosis such as transbronchial needle aspiration under endobronchial ultrasound guidance (EBUS-TBNA) and endobronchial biopsy (EBB) were not included in the diagnostic process in this study. The extent to which TBLC in combination with EBUS-TBNA and EBB can contribute to a further increase in diagnostic confidence needs to be investigated in a separate prospective study. However, the different diagnostic value of TBLC and TBLF alone must be considered for cases in which EBUS-TBNA and EBB cannot confirm a histological diagnosis.

## 5. Conclusions

In this prospective study, the diagnosis of sarcoidosis was made more frequently with TBLC than with TBLF, although with a higher risk of bleeding. The use of TBLC should be considered in bronchocentric ILD in addition to other parenchymal ILDs.

## Figures and Tables

**Table 1:** Bronchoscopic intervention.

Bronchoscopic Intervention	TBLC (*N* = 17)	TBLF (*N* = 17)	*p*-Value
Biopsy procedure			
Number of biopsies—no. (%) Total number Number per patient—mean	61 3.6 (±1.2)	68 4.0 (±1.3)	n.s.
Size of biopsy probe ^†^—no. (%) Small Large	10 (62.5) 6 (37.5)	8 (47.0) 9 (52.9)	n.s.
Biopsy location—no. (%) Upper lobe—right/left Middle lobe/Lingula Lower lobe—right/left	19 (31.1)/5 (8.2) 2 (3.3)/6 (9.8) 25 (41.0)/4 (6.6)	23 (33.8)/10 (14.7) 3 (4.4)/4 (5.9) 23 (33.8)/5 (7.4)	n.s.
Positioning of biopsy probe—no. (%) Easy Intermediate Difficult	10 (58.8) 7 (41.2) 0 (0.0)	14 (82.3) 3 (17.6) 0 (0.0)	n.s.
Duration of biopsy technique—mean (min)	8.0 (±6.1)	5.6 (±4.5)	
Biopsy sample characteristics			
Area of biopsies Total area of all biopsies—median (mm^2^) Area per biopsy—median (mm^2^)	26.07 8.11	8.19 1.72	*p* < 0.05
Artefacts (percentage of area) no. (%) No artefacts 0–10% 11–20% >20%	13 (76.5) 3 (17.6) 1 (5.9) 0 (0.0)	4 (23.5) 5 (29.4) 6 (35.3) 2 (11.8)	*p* < 0.05

Values show absolute numbers and percentages; plus–minus values means ± standard deviation (SD). Cryoprobe size was unknown in one patient, and biopsy duration for TBLC and TBLF in one patient. TBLC—transbronchial lung cryobiopsy; TBLF—transbronchial lung forceps biopsy. ^†^ Small: TBLC 1.9 mm, TBLF 1.8–2.0 mm; Large: TBLC 2.4 mm, TBLF 2.2–2.6 mm.

**Table 2:** Histological diagnosis of sarcoidosis by biopsy.

Diagnosis of Sarcoidosis in the Overall Cohort		TBLC 272 (100)	
		Yes 16 (5.9)	No 256 (94.1)
TBLF 272 (100)	Yes 7 (2.6)	6 (2.2)	1 (0.4)
	No 265 (97.4)	10 (3.7)	255 (93.8)
		*p* < 0.0001	

Distribution of sarcoidosis cases, according to biopsy technique and sarcoid diagnosis outcome. no (%)—absolute number and percentage; TBLC—transbronchial lung cryobiopsy; TBLF—transbronchial lung forceps biopsy.

**Table 3:** Adverse bleeding events.

Adverse Bleeding Events		TBLC (*N* = 17)	TBLF (*N* = 17)	*p*-Value
Clinical relevance	Bleeding severity			
Low	No	7 (41.1)	8 (50.0)	n.a.
	Mild	7 (41.1)	8 (50.0)	
High	Moderate	2 (11.8)	0 (0.0)	
	Severe	1 (5.9)	0 (0.0)	

Bleeding severity was unknown in one patient for TBLF. n.a.—not applicable because of 0 cases in the TBLF high clinical relevance group.

## Data Availability

The data presented in this study are available on request from the corresponding author.
